# Synergistic Strategy for Multicolor Two-photon Microscopy: Application to the Analysis of Germinal Center Reactions In Vivo

**DOI:** 10.1038/s41598-017-07165-0

**Published:** 2017-08-02

**Authors:** Asylkhan Rakhymzhan, Ruth Leben, Hanna Zimmermann, Robert Günther, Peggy Mex, David Reismann, Carolin Ulbricht, Andreas Acs, Alexander U. Brandt, Randall L. Lindquist, Thomas H. Winkler, Anja E. Hauser, Raluca A. Niesner

**Affiliations:** 1 Deutsches Rheumaforschungszentrum, a Leibniz Institute, Berlin, Germany; 2 Nikolaus-Fiebiger-Zentrum, Division of Genetics, Department of Biology, University of Erlangen-Nürnberg, Erlangen, Germany; 3 Immundynamics, Charité – Universitätsmedizin Berlin, Berlin, Germany; 4 NeuroCure Clinical Research Center, Clinical and Experimental Multiple Sclerosis Research Center, Department of Neurology, Charité – Universitätsmedizin Berlin, Berlin, Germany

## Abstract

Simultaneous detection of multiple cellular and molecular players in their native environment, one of the keys to a full understanding of immune processes, remains challenging for *in vivo* microscopy. Here, we present a synergistic strategy for spectrally multiplexed *in vivo* imaging composed of (i) triple two-photon excitation using spatiotemporal synchronization of two femtosecond lasers, (ii) a broad set of fluorophores with emission ranging from blue to near infrared, (iii) an effective spectral unmixing algorithm. Using our approach, we simultaneously excite and detect seven fluorophores expressed in distinct cellular and tissue compartments, plus second harmonics generation from collagen fibers in lymph nodes. This enables us to visualize the dynamic interplay of all the central cellular players during germinal center reactions. While current *in vivo* imaging typically enables recording the dynamics of 4 tissue components at a time, our strategy allows a more comprehensive analysis of cellular dynamics involving 8 single-labeled compartments. It enables to investigate the orchestration of multiple cellular subsets determining tissue function, thus, opening the way for a mechanistic understanding of complex pathophysiologic processes *in vivo*. In the future, the design of transgenic mice combining a larger spectrum of fluorescent proteins will reveal the full potential of our method.

## Introduction

Multiplex fluorescent imaging as well as non-imaging techniques are widely used to follow immune responses in space and time. Most *ex vivo* studies are performed on fixed, static samples, making repeated investigations of the same sample possible by serial detection. Hence, established methods such as flow cytometry (FACS)^1^ or immunofluorescence-based confocal microscopy^2^ are capable of recording 10 or more parameters by using sequential excitation of differentially excited fluorophores. Multi-epitope-ligand cartography^3^ (MELC) allows even more parameters to be investigated, over 100 within one sample, by using multiple cycles of labeling, detection, and bleaching. Although such approaches are appropriate for *ex vivo* investigations of fixed tissue, they are not suitable for intact, live tissue, which is highly dynamic on both the cellular and molecular levels. Referring especially to MELC, the great multiplexing potential but time-consuming acquisition can be used only as a preliminary step for a full understanding of the complex cellular interplay during immune responses. *In vivo* spectrally multiplexed imaging approaches are needed to visualize and understand the dynamic dimensions of pathophysiologic phenomena.

Over the last decades, *in vivo* two-photon microscopy has become the main tool for imaging cellular responses in live animals, with particular application in neuroscience and immunology^4–10^. The inherent optical sectioning, high penetration depth and low phototoxicity of two-photon microscopy allow visualization of cellular dynamics in space and time, in their natural environment *in vivo*. For instance, the dynamic processes taking place during the germinal center (GC) reaction are highly relevant to understanding mechanisms involving the maturation of B cell immune responses. *In vivo* imaging of GCs has allowed quantitative description and modeling of B cell motility patterns as well as communication between antigen-specific B cells and T follicular helper (Tfh) cells and interactions of B cells with follicular dendritic cells (FDC)^5, 10^. However, current *in vivo* techniques allow for simultaneous observation of typically 3 to 4 fluorophores^11–14^ in addition to second and/or third harmonics generated from organized structures like collagen. This is not enough to monitor the communication and interplay of all cellular and tissue compartments involved in a germinal center reaction, as GC B cells, naive B cells, Tfh cells and FDCs must all be visualized, leaving no channels for reporters of signaling, clonality, cell division, or cell fate. How antigen-presenting cells, such as dendritic cells, communicate with T cells, how germinal center B cells in contrast to naïve B cells interact with T cells and/or with FDCs, how the selected germinal center B cells egress as plasma blasts or memory B cells from the germinal center and how the vasculature is involved in all these processes – all these phenomena have been studied in detail, however each in separate experiments. The interactions between GC B cells, Tfh cells, and FDCs also result in many signaling events which have also been imaged, but in isolation, with one signaling event per experiment. Only their correlated investigation in one and the same germinal center will allow conclusions on the spatiotemporal sequence of their occurrence, giving a more complete picture of the adaptive immune response.

The quantitative limit for simultaneous visualization of multiple fluorophores in current intravital microscopy can be overcome by solving three main challenges^15^: optimization of the excitation scheme, extension of the fluorescence range using far-red and near-infrared (NIR) probes, and improving the effectiveness of spectral unmixing.

For *in vivo* imaging, it is critical to simultaneously image all fluorophores. As cells move dynamically, sequential excitation of large volumes will lead to difficulties in synchronizing images acquired at different times. This requires efficient simultaneous excitation over the entire range of fluorophores, which is easily performed with relatively inexpensive continuous-wave lasers, but is difficult to achieve with the femtosecond-pulsed laser sources optimal for two-photon excitation. Different excitation schemes in two-photon microscopy were used to visualize multiple fluorophores in live animals by us and others: sequential single excitation by Ti:Sa laser^14^, dual excitation by a Ti:Sa laser and an optical parametric oscillator (OPO)^11, 12^, and triple excitation using wavelength mixing of Ti:Sa and OPO, leading to two-color-two-photon excitation^13^. The two-color two-photon excitation using picosecond or even femtosecond lasers was first demonstrated as the wavelength mixing of 800 nm and its second harmonic of 400 nm on laser dyes (p-therphenyl, 2-methyl-5-tert-butyl-p-quaterphenyl) and on tryptophan^16, 17^.

Thanks to recent developments of nonlinear fiber optics^18^ and ceramic-based electro-optic intensity modulators^19^, cheaper alternative optical sources have become available for two-photon excitation, which are in principle applicable to sets of multiple fluorophores. The invention of photonic crystal fibers (PCF) in the 1990s^20, 21^ stimulated a rapid development of optical sources with extreme spectral broadening, termed supercontinuum (SC) generation. Due to the broad spectral bandwidth, a fiber SC offers simultaneous access to multiple wavelengths in a uniform spatial profile of a single-wavelength laser^22^. Furthermore, another alternative for multiple fluorophore excitation was achieved by using fast wavelength modulation based on ceramic PMN-PT intensity modulators and soliton self-frequency shift in nonlinear PCF^19^. This system switches wavelength of a 100 fs pulsed laser source within the range of 200 nm in approximately 5 μs, which is problematically long for point-scanning systems. While these excitation sources have great potential, their application with common fluorescent proteins *in vivo* needs to be better characterized.

The near infrared (NIR) region, known as the optical window, is optimal for deep imaging in live mammals, as it has relatively low scattering, reduced autofluorescence and high tissue transparency, due to low absorption of water and haemoglobin at these wavelengths^23^. Recent developments in the field of NIR fluorescent proteins^24, 25^ have extended the detection range of fluorescent probes, resulting in an increased number of available fluorophores for *in vivo* imaging experiments, and the longer emission wavelengths of these proteins should permit imaging deeper into tissue.

Spectrally multiplexed imaging implies not only the effective excitation and detection of multiple fluorophores, but also their unambiguous discrimination. In order to distinguish between multiple fluorophore signals, a large variety of spectral unmixing methods have been developed. Linear spectral unmixing is a simple, widely used method available both in commercial (Zeiss, Nikon) and in open-source software (Fiji/ImageJ, J. Walter PlugIn). The algorithm is based on the assumption that the detection signal is linearly dependent on the contributing fluorophores^26, 27^. Alternatively, spectral deconvolution was applied to separate six different signals in the brain of triple transgenic mice by using sequentially collected hyper-stacks of fluorophore signatures^14^. Combining color balancing^28^ and signal subtraction from adjacent channels allowed tumor cell lines expressing one of five different fluorophores to be distinguished in immunodeficient mice^11^. As scattering and wave front distortions are extremely nonlinear with respect to wavelength, we expect the tissue environment to change the spectra of the contributing fluorophores nonuniformly. In this case, the linear unmixing approach cannot isolate different components contributing to the emitted signal. Better separation quality may be achieved by using similarity approaches, which rely on reference spectra acquired *in situ* ^29^. Further, current unmixing approaches do not allow the detection of more fluorophores than the available detection channels, in simultaneously acquired imaging data.

We demonstrate simultaneous spectrally multiplexed detection of seven fluorophore signals corresponding to seven cellular and tissue compartments in popliteal lymph nodes of live mice: naïve B cells, CD4+ T cells, antigen-specific B1-8 germinal center B cells, plasmablasts, follicular dendritic cells, blood vessels and macrophages, including tingible body macrophages. To achieve this aim, we combined: (i) wavelength mixing allowing for effective simultaneous triple two-photon excitation of fluorophores, (ii) extension of the fluorescence detection range by using far-red and NIR fluorophores and, (iii) effective non-analytic spectral unmixing. As an effective color discrimination technique, we developed a new algorithm based on the principle of similarity unmixing, called SIMI. This algorithm stays fully functional beyond the fundamental limit of linear unmixing, where the number of fluorophores cannot exceed the number of detection channels. Using this novel imaging strategy, we were for the first time able to investigate the dynamics of naïve B cells, antigen-specific B cells, CD4+ T helper cells, follicular dendritic cells and tingible body macrophages as well as the immediate egress of plasma blasts during ongoing GC reactions, while highlighting the vasculature as the site of cellular exchange between different organs. Our technique is a versatile tool able to open new insights into mechanisms of complex dynamic immune processes *in vivo*, applicable to the investigation of any organ, in which the communication of various cell subtypes defines tissue function and dysfunction.

## Results

### Two-photon microscope setup for simultaneous triple excitation using wavelength mixing

**Figure 1:**
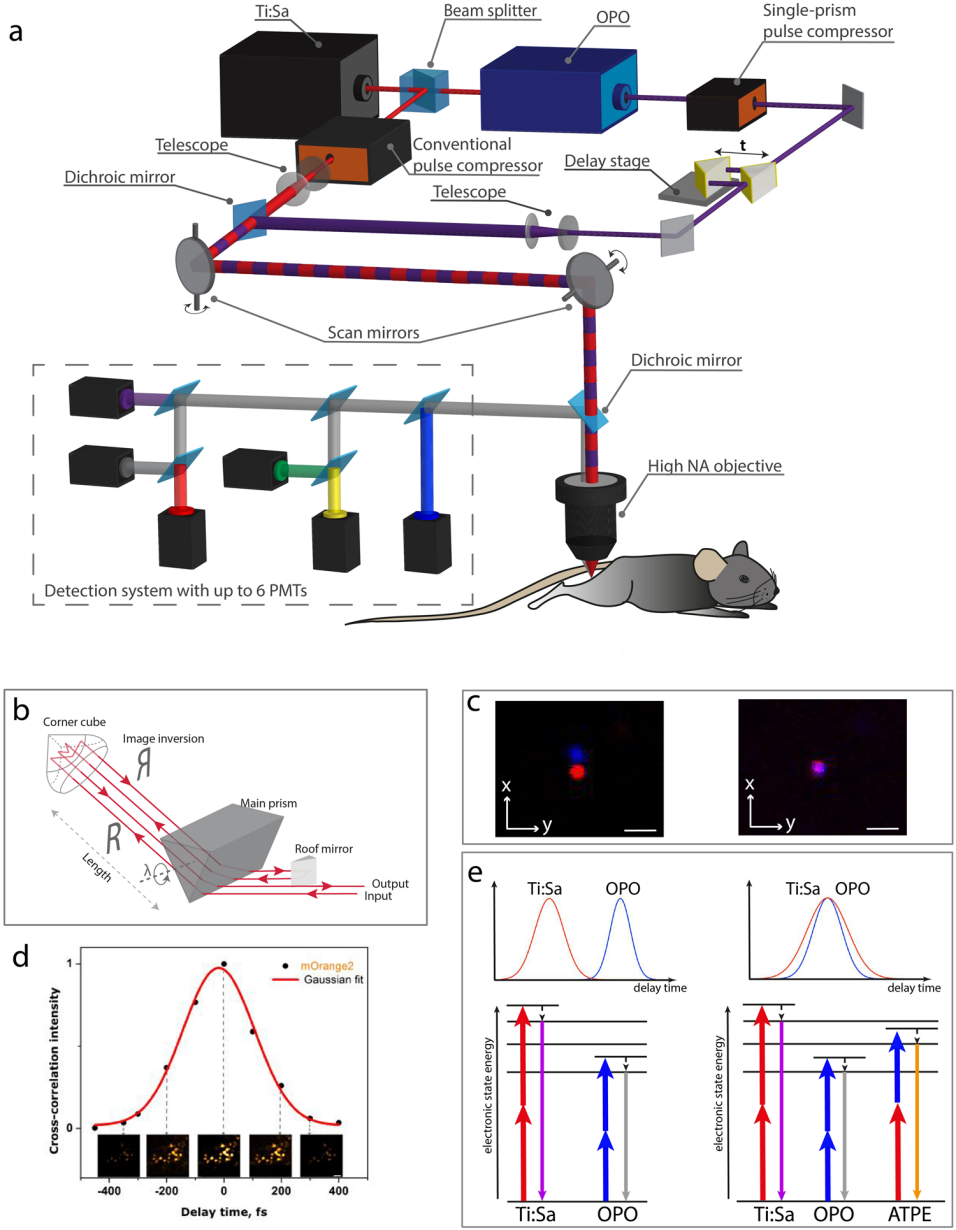
Principle of wavelength mixing two-photon microscopy: setup and characterization. (**a**) Imaging setup. A femtosecond laser (Ti:Sa) beam is divided in two fractions by a beam splitter. One is adjusted in a microscope, the second pumps an OPO. Pulses from Ti:Sa and OPO are synchronized using a delay stage (t) and spatially coaligned. GVD compensation is achieved using a conventional prism-based and a single-prism pulse compressors for Ti:Sa and OPO, respectively. The relative divergence of two laser beams is controlled using telescopes. Galvanoscan mirrors provide raster scanning the area up to 500 × 500 um^2^. Chromatic aberration between NIR and IR wavelength of two lasers is corrected by the high numerical aperture objective. The non-descanned detection system is equipped with six PMTs. (**b**) Single-prism pulse compressor. Negative dispersion is accumulated on the four-pass travel through the main prism. The distance between a corner cube and the main prism is half that of the conventional design due to the image inversion of the corner cube. Pulse compression at different wavelengths can be achieved by rotating only one prism. (**c**) Spatially separated (left) and overlapping (right) Ti:Sa (red) and OPO (blue) foci in the microscope, measured on a 100 nm fluorescent bead (λ_emission_ = 605 nm). Scale bars, 0.2 μm. (**d**) Optimization of the ATPE. The mOrange2 fluorescent signal can be independently controlled by adjusting the delay between pulses. The insets represent images of HEK cells expressing mOrange2 at different delay times. Scale bar, 50 μm. (**e**) Spatiotemporal overlapping of two laser pulses. Left side: unsynchronized pulses (850 nm and 1230 nm) provide dual two-photon excitation, i.e. two parallel symmetric two-photon excitation processes. Right side: The wavelength mixing appears only if the pulses are synchronized in time and the two foci are matched in space. A third, asymmetric two-photon excitation process additionally takes place, making further fluorophores visible. Hence, simultaneous triple two-photon excitation of a broad set of chromophores is achieved.

In order to achieve effective triple excitation in a two-photon microscope, for dynamic, spectrally multiplexed intravital imaging, we optimized a conventional setup, as described in the following (Fig. 1a). The excitation system of our microscope consists of two laser sources: a femtosecond Ti:Sa laser and an optical parametric oscillator (OPO) pumped by the Ti:Sa (Fig. 1a). Spatial overlap of OPO and Ti:Sa beams in the microscope allows simultaneous dual two-photon excitation of fluorophores^6^. We achieved optimal spatial overlap of the two laser sources by imaging sub-diffraction nano-spheres of 100 nm diameter and 605 nm emission, excitable by both lasers (Fig. 1c). In addition, we synchronized the pulse trains of Ti:Sa and OPO in time, using a customized delay stage as shown in Fig. 1a. The delay stage is positioned on the optical path of the OPO and consists of two 90° prisms, with broad-band reflecting catheti. One prism is fixed, the other is placed on a piezo stage with a translation step of 15 nm. Thus, by moving the piezo stage, the optical path length of the OPO can be varied with an accuracy of 50 attoseconds (0.05 fs). In this way, OPO and Ti:Sa pulse trains can be adjusted to perfectly overlap, since Ti:Sa and OPO have the same repetition rate, as the OPO is being pumped by the Ti:Sa. We verified synchronization of the pulse trains by measuring sum frequency generation (SFG) signals in a powder of birefringent potassium dihydrogen phosphate (Suppl. Video 1).

Spatial foci overlap of the two lasers in the microscope enables dual two-photon excitation originating from the individual laser sources, i.e. only Ti:Sa and only OPO – results in symmetric two-photon excitation processes. The additional time overlap of the pulse trains allows for a third excitation process: two-photon excitation triggered by the combination of one Ti:Sa and one OPO photon (Fig. 1e). This process represents an asymmetric two-photon excitation (ATPE) and is a wavelength mixing process of the two lasers in non-linear medium, similar to SFG. ATPE is equivalent to a two-photon excitation with a virtual wavelength λ_3_ = 2/(1/λ_1_ + 1/λ_2_), where λ_1_ is the wavelength of Ti:Sa and λ_2_ is the wavelength of OPO. We optimized this wavelength mixing configuration to effectively excite various blue and green emitting fluorophores by Ti:Sa (λ_1_ = 850 nm), far-red and near-infrared emitting fluorophores by OPO (λ_2_ = 1230 nm), and orange and red emitting fluorophores by ATPE (virtual λ_3_ = 1005 nm). Hence, triple two-photon excitation of a broad range of fluorophores (Fig. 1e) and independent control of orange and red fluorophore signal are possible (Fig. 1d, Suppl. Video 2).

In order to perform triple two-photon excitation in an effective manner, a maximum ratio of peak to average power of both Ti:Sa and OPO is needed. Therefore, it is crucial to control the pulse widths of not only both lasers, but also of their wavelength mixing. Propagation through dispersive optical elements, such as animal tissue, causes significant broadening of femtosecond pulses due to group velocity dispersion (GVD). To counteract this, we used external prism-based pulse compressors for both the Ti:Sa and the OPO. For the Ti:Sa beam, a conventional pulse compressor^30^ with a two-prism configuration was already integrated in our setup. In order to mitigate GVD in the OPO beam path, we built a compact single-prism pulse compressor^31^. The single-prism design consists of a main prism, a roof mirror and a corner cube (Fig. 1b). Negative dispersion of the pulses is achieved on the four-pass travel from the main prism to the corner cube, reducing the nominal distance in the pulse compressor to the half as compared to conventional “two-prism” pulse compressors. The use of only one prism and the optical properties of the corner cube simplify compensation of different spatiotemporal distortions in the output beam as well as alignment of the setup (*Suppl. Material*). Using a beam auto-correlator, we found that the OPO pulse width without GVD compensation increases from 190 fs at the laser output to 340 fs in the microscope. Our single-prism compressor narrows down the pulse duration to 160 fs at the focus of the microscope. The pulse width of the Ti:Sa beam in the microscope is ~250 fs after GVD compensation (Suppl. Figure 1). Finally, the cross-correlation pulse width of the wavelength mixing of 850 nm (Ti:Sa) and 1230 nm (OPO) as measured by ATPE of mOrange2 amounts to ~250 fs (Fig. 1d). All pulse width values were calculated by approximating the autocorrelation and cross-correlation curves with Gaussian functions. The peak intensity values at 10 mW average power at the focal plane (objective lens NA = 1.0) amount to 2.34·10^28^ photon/cm^2^·s at 850 nm (Ti:Sa), 2.47·10^28^ photon/cm^2^·s at 1230 nm (OPO) and 2.77·10^28^ photon/cm^2^·s at virtual the 1005 nm for ATPE and SFG. The total real photon flux at the sample surface amounts to 4.81·10^28^ photon/cm^2^·s, independent of the lasers wavelengths (in total approx. 20 mW average power). The dwell time of both lasers within one focus of the sample did not exceed 4 µs to avoid photobleaching and photodamage. Thus, the optical premises for effective, simultaneous triple two-photon excitation of fluorophores with emission spectra ranging from blue to near-infrared and its use in dynamic live imaging are given.

### Spectrally multiplexed imaging of live cells by simultaneous triple excitation of up to six chromophores

**Figure 2:**
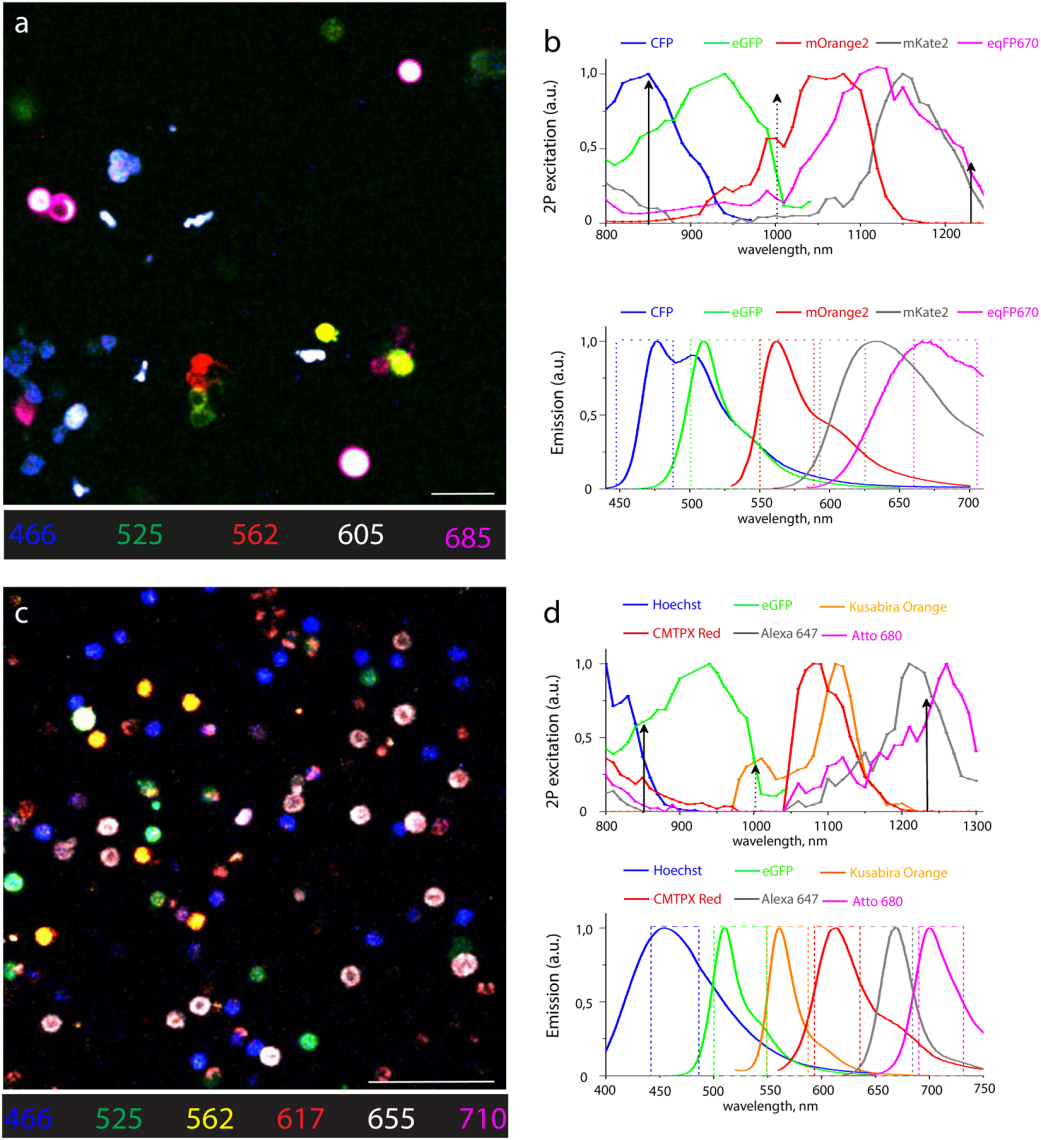
*In vitro* spectrally multiplexed imaging of five fluorescent proteins in HEK cells and six chromophores in murine splenocytes. (**a**) Raw fluorescence image of a mixture of HEK cells singly expressing one of five FPs: CFP, eGFP, mOrange2, mKate2, and eqFP670. (**b**) Two-photon excitation and emission spectra of CFP, eGFP, mOrange2, mKate2, and eqFP670. Two-photon spectra of mOrange2, mKate2, and eqFP670 were recorded in this work (Methods). Arrows indicate effective excitation wavelengths and rectangles indicate the filter bandwidth of the five detection channels (a.u., arbitrary units). (**c**) Raw image of a mixture of murine splenocytes each labelled with one of six fluorophores: Hoechst, eGFP, Kusabira Orange, CMTPX Red, Alexa 647, and Atto 680. (**d**) Two-photon excitation and emission spectra of Hoechst, eGFP, Kusabira Orange, CMTPX Red, Alexa 647, and Atto 680. 2P spectra of Hoechst, Kusabira Orange, CMTPX Red, Alexa 647, and Atto 680 were recorded (Methods). Arrows indicate optimal excitation wavelengths and rectangles indicate the filter bandwidth of the six detection channels (a.u., arbitrary units). Scale bars, 50 μm.

Using our setup, we demonstrated spectral multiplexing on mixtures of isolated cells either expressing fluorescent proteins or labeled by dyes for live imaging. We chose two different *in vitro* models, which can be easily translated to *in vivo* imaging experiments. First, we imaged a mixture of human embryonic kidney (HEK-293T) cell lines, each expressing one out of five fluorescent proteins: eCFP, eGFP, mOrange2, mKate2 and eqFP670 (Fig. 2a). This model resembles features of Brainbow3.0 or Confetti mice, which have been used in live animal imaging to monitor clonal relationships on the cellular level in various tissues^32, 33^. eGFP, mOrange2 and mKate2 were chosen as the core of the Brainbow3.0 transgenic line due to their high photo-stability, minor tendency to aggregate *in vivo*, low sequence homology and minimal spectral overlapping^32^. In order to extend the emission spectrum of the model (and its application range), we added two more proteins, eCFP and eqFP670. The eqFP670 is a NIR fluorescent protein based on the dimeric far-red fluorescent protein Katushka-9-5^25^, characterized by high photo- and pH-stability, outstanding brightness and low cytotoxicity.

Secondly, we imaged isolated murine splenocytes containing one out of six fluorophores (Fig. 2c), commonly used in multiplexed intravital imaging of various cell subsets during immune reactions. The six fluorophores included both dyes, i.e. Hoechst, CMTPX Red, Alexa647 and Atto680, as well as the fluorescent proteins eGFP and monomeric Kusabira Orange. We chose these fluorophores based on their high photostability and brightness, low cytotoxicity, and minimal spectral overlap.

In order to ensure optimal triple excitation of the chosen chromophores, both fluorescent proteins and dyes, we measured their two-photon excitation spectra in cells, as previously described^12^ (Fig. 2b,d; upper panels). Spectra were acquired in live cells to allow for better comparability with *in vivo* preparations. Thus, we found that wavelength mixing of 850 nm (Ti:Sa) and 1230 nm (OPO) pulses is the optimal combination for efficient simultaneous excitation of both the five fluorescent proteins expressed by HEK cells and of the six chromophores used to label splenocytes (Fig. 2a,c).

Based on the emission spectra of our selected fluorophores (Fig. 2b,d; lower panels), we defined an optical system of dichroic mirrors and interference filters to distribute the emitted signal on the six PMT detectors (Fig. 1a), in order to achieve optimal spectral resolution of the chromophores. However, despite this optimized spectral separation of the detected signal, significant crosstalk is observed between several chromophores, thereby preventing their unambiguous spectral separation and detection. This shortcoming can only be resolved by post-processing of the imaging data, using specialized spectral unmixing approaches, as described in the next section.

### SIMI – similarity unmixing approach for optimal post-processing chromophore resolution in imaging data

Any spectral unmixing procedure represents a transformation of the image from the “detection channel space” to the “fluorophore space”. In order to distinguish between chromophores excited by simultaneous triple two-photon excitation, we developed a new algorithm of spectral unmixing named similarity unmixing (SIMI). Starting from the concept of similarity between reference spectra and the measured signal^23^, the SIMI approach is a numerical pixel-based method, which separates mixed colors based on similarities between overlapping fluorophores as well as on the spectral fingerprints of the individual fluorophores (spectral signatures). The SIMI approach originates from the widely-used linear unmixing method, but it does not follow its algebraic solving strategy. Thus, it enables the simultaneous identification of a higher number of chromophores than available detector channels from imaging data.

The linear unmixing algorithm is based on the assumption that the total signal *S*_*i*_ measured on every detection channel is linearly proportional to the combination of contributing fluorophores *F*_*j*_^34^:

1 $${S}_{i}={a}_{1i}\times {F}_{1}+{a}_{2i}\times {F}_{2}+\cdots =\sum _{i=1}^{m}{a}_{ij}{F}_{j},\quad i=1\div m$$

or in matrix form:

2 $$(\begin{array}{c}{S}_{1}\\ \vdots \\ {S}_{n}\end{array})=[\begin{array}{ccc}{a}_{11} & \cdots  & {a}_{1m}\\ \vdots  & \ddots  & \vdots \\ {a}_{n1} & \cdots  & {a}_{nm}\end{array}]\times (\begin{array}{c}{F}_{1}\\ \vdots \\ {F}_{m}\end{array}),$$

where *α*_*ij*_ is the mixing matrix element, *i* is the channel index, *j* is the fluorophore index, *n* is the number of detection channels, *m* is the number of fluorophores. The principle of linear unmixing consists in finding the vector *F*_*j*_ by calculating the contribution values of the given fluorophores, i.e. by algebraically solving the system of linear equations (1) or (2)^26, 27^. The matrix equation (2) may be also written as^35^:

3 $$(\begin{array}{c}{S}_{1}\\ \vdots \\ {S}_{n}\end{array})={F}_{1}\cdot (\begin{array}{c}{a}_{11}\\ \vdots \\ {a}_{n1}\end{array})+{F}_{2}\cdot (\begin{array}{c}{a}_{12}\\ \vdots \\ {a}_{n2}\end{array})+\cdots +{F}_{m}\cdot (\begin{array}{c}{a}_{1m}\\ \vdots \\ {a}_{nm}\end{array}),$$

where it can be interpreted in terms of fluorophore components or fingerprints. The column ${a}_{1j}\ldots {a}_{nj}$ on the right side of the equation (3) represents the fingerprint of the fluorophore *F*_*j*_, satisfying the normalization condition $\sum _{i=1}^{m}{a}_{ij}=1$, for *i* = 1÷ *m*. Each fingerprint can be defined from the normalized signals ${c}_{1}\ldots {c}_{n}$ measured at single color condition, when only one fluorophore is present, *F*_*j*_ ≠ 0 and ${F}_{j\ne k}=0$, for $k=1\div m$. Thus, equation (3) becomes:

4 $$(\begin{array}{c}{S}_{1}\\ \vdots \\ {S}_{n}\end{array})={S}_{max}\cdot (\begin{array}{c}{c}_{1}\\ \vdots \\ {c}_{n}\end{array})={F}_{j}\cdot (\begin{array}{c}{a}_{1j}\\ \vdots \\ {a}_{nj}\end{array}),$$

with *S*_*max*_ and *F*_*j*_ as constant values. The fingerprint elements ${a}_{1j}\ldots {a}_{nj}$ represent a relative intensity distribution, which reflects the emission spectrum of fluorophore *F*_*j*_.

SIMI algorithm assigns an unknown fluorescence signal fingerprint to a fluorophore by comparing and finding the closest match between the normalized signal column ${b}_{1j}\ldots {b}_{nj}$ measured from the images with mixed fluorophores and the fingerprint elements ${a}_{1j}\ldots {a}_{nj}$ obtained from images of single fluorophores:

5 $$(\begin{array}{c}{S}_{1}\\ \vdots \\ {S}_{n}\end{array})={S}_{max}\cdot (\begin{array}{c}{b}_{1}\\ \vdots \\ {b}_{n}\end{array})\leftrightarrow {F}_{1}\cdot (\begin{array}{c}{a}_{11}\\ \vdots \\ {a}_{n1}\end{array}),{F}_{2}\cdot (\begin{array}{c}{a}_{12}\\ \vdots \\ {a}_{n2}\end{array}),\ldots ,{F}_{m}\cdot (\begin{array}{c}{a}_{1m}\\ \vdots \\ {a}_{nm}\end{array}).$$

The matching procedure was performed by a gradient fitting approach that minimizes the square difference of the signal *b*_*i*_ and the fingerprint *a*_*ij*_ values:

6 $${R}_{j}^{2}=\sum _{i}{({b}_{i}-{a}_{ij})}^{2}.$$

Owing to the fact that our algorithm defines the degree of correlation, or similarity, but not the contribution value of the mixed fluorophores, the correct assignment of the mixed colors can be achieved only if each cell contains only one chromophore, i.e. one-fluorophore-per-cell condition.

**Figure 3:**
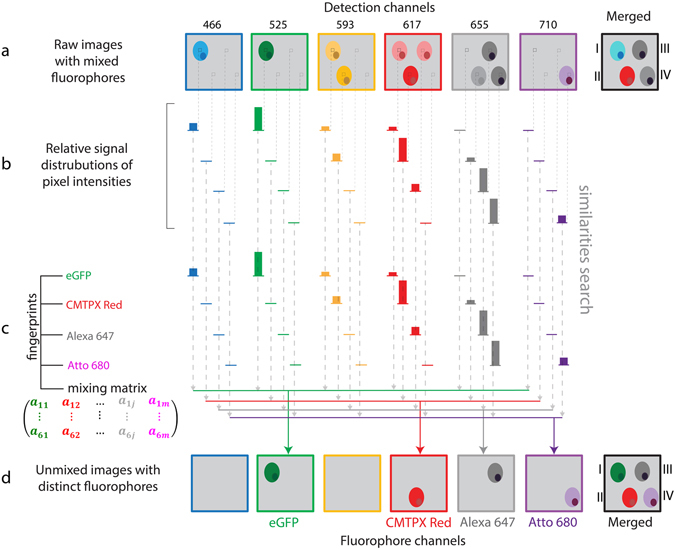
Principle of similarity unmixing algorithm. (**a**) Raw images of cellular objects show crosstalk in six detection channels. The merged image illustrates color ambiguity of four cells labelled from I to IV. (**b**) The algorithm extracts a relative signal distribution from six channels pixel by pixel. (**c**) The fingerprints of eGFP, CMTPX Red, Alexa647, Atto680 are measured from single-labeled cells. The algorithm searches for similarities between the signatures of undefined fluorophores from (**b**) and the known fingerprints channel by channel (dashed arrows). (**d**) Unmixed images with separated distinct fluorophores. The closest match with one of the fingerprints indicates similarity of the undefined fluorophore from (**c**) with the fluorophore of this fingerprint. The determined fluorophore signal is collected in the distinct fluorophore channel (colored arrows). As a result, color separation of the cells I–IV is achieved in the merged image from the fluorophore channels.

We implemented the SIMI algorithm into the spectral unmixing PlugIn of Fiji/ImageJ (J. Walter). The SIMI procedure is schematically shown for the detection system with six channels in Fig. 3. Undefined fluorophore signals from four cell types show color mixing caused by bleed-through into neighboring channels (Fig. 3a). First, the algorithm extracts the normalized signal distribution over channels ${b}_{1}\ldots {b}_{n}$ according to equation (5) in each pixel. Individual fluorophores exhibit a signal distribution corresponding to their emission spectra (Fig. 3b). Next, the algorithm searches for similarities between the normalized distribution ${b}_{1}\ldots {b}_{n}$ and the measured fingerprints ${a}_{1j}\ldots {a}_{nj}$ in equation (4) (dashed arrows in Fig. 3b,c). The closest match with one of the fingerprints identifies the signal origin and, thus, the fluorophore. Finally, the identified signal is displayed in a dedicated channel (Fig. 3d). As a result, the four cells have the closest match with the fingerprints of eGFP, CMTPX Red, Alexa647 and Atto680, respectively, and are placed in the corresponding fluorophore channels (Fig. 3d). The merged image of the fluorophore channels shows complete color unmixing. The cell pairs I–II and III–IV represent different levels of color mixing. Cells I and II show clear domination of the green and red channels, respectively. Color balance correction can easily assign these cells to eGFP and CMTPX Red expression, as seen on the merged image in Fig. 3a. In contrast, cells III and IV have their maximum signal in the same channel (grey) and are hardly distinguishable. To identify these cells, the information from all channels needs to be used. The SIMI algorithm is able to separate cells III and IV based on the signal difference in the red and magenta detection channels.

**Figure 4:**
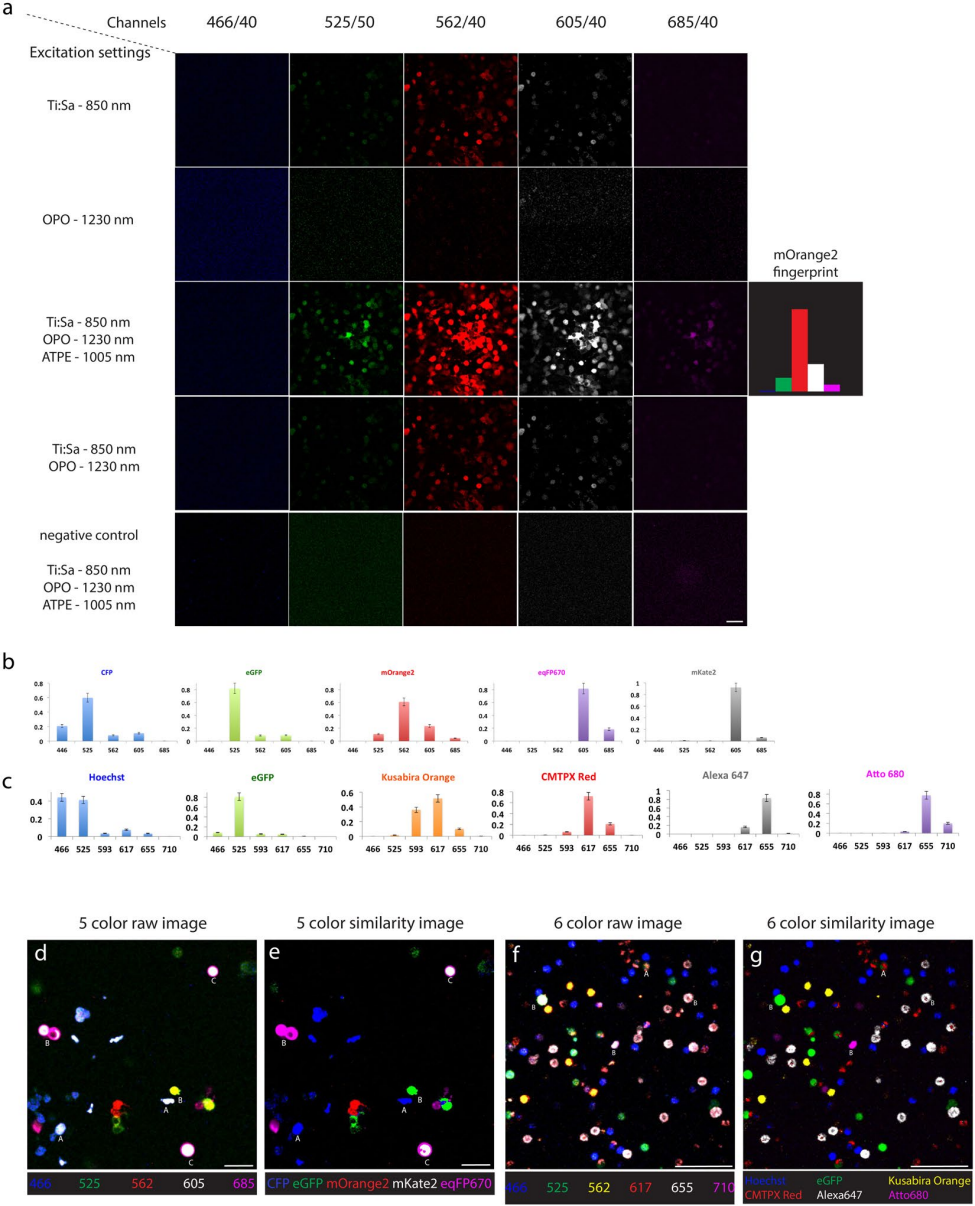
Similarity unmixing of spectrally multiplexed images of isolated cells. (**a**) Single color measurement of HEK cells expressing only mOrange2. Rows represent four excitation schemes: single 2P excitation at 850 nm by Ti:Sa laser; single 2P excitation at 1230 nm by OPO; the wavelength mixing 2P excitation at 850 nm, 1005 nm and 1230 nm by Ti:Sa laser, ATPE and OPO laser, respectively; dual unsynchronized 2P excitation at 850 nm and 1230 nm by Ti:Sa and OPO, respectively. The columns display images from five detection channels. The elements of the fingerprints are calculated as average signal values at the wavelength mixing scheme. (**b**) The fingerprints of five FPs, CFP, eGFP, mOrange2, mKate2, and eqFP670. (**c**) The fingerprints of six fluorophores, Hoechst, eGFP, Kusabira Orange, CMTPX Red, Alexa 647, and Atto 680. (**d**,**e**) Raw and unmixed images of HEK cell mixture expressing one of five FPs. Letter A indicates example of the crosstalk of the overexpressed signal from minimal spectrally overlapped FPs (case I and II cells in Fig. 3). Letter B indicates example of the crosstalk from strong spectral overlap (case III and IV in Fig. 3). Letter C indicates example of both crosstalk effects, signal overexpression and strong spectral overlapping. (**f**,**g**) Raw and unmixed images of splenocyte mixture expressing six fluorophores at one-fluorophore-per-cell condition. Letter A indicates example of the crosstalk of the overexpressed signal from minimal spectrally overlapped fluorophores (case I and II cells in Fig. 3). Letter B indicates example of the crosstalk from strong spectral overlap (case III and IV in Fig. 3). Scale bars, 50 μm.

In order to determine the fingerprint of each fluorophore in our *in vitro* models, we imaged live cells containing only a single fluorophore, either a fluorescent protein or dye, as shown in the example of HEK-293T cells expressing mOrange2 (Fig. 4a). Each row corresponds to one of four excitation schemes: excitation only by Ti:Sa, excitation only by OPO, triple excitation by wavelength mixing of Ti:Sa and OPO, and dual excitation by temporally non-synchronized Ti:Sa and OPO. In HEK cells expressing one of five fluorescent proteins: eCFP, eGFP, mOrange2, mKate2 and eqFP670, mOrange2 was mostly excited by ATPE as highlighted by the difference between the third and fourth row in Fig. 4a. The last row in Fig. 4a illustrates a negative control of HEK cells without any of our fluorescent proteins. The broad signal distribution from green to magenta channels represents the fingerprint of mOrange2, which is defined as the normalized intensity histogram calculated from the average signal intensities of images in all detection channels. In this way, we determined the fingerprints of all chromophores used in this study (Fig. 4b,c; Suppl. Figs 2 and 3) and applied them to distinguish the images acquired in mixtures of HEK cells (Fig. 4d,e) and splenocytes (Fig. 4f,g). Taken together, while color balance correction is able to resolve fluorophores with strongly differing fingerprints, SIMI provides spectral unmixing for spectral signatures with subtle differences.

### SIMI algorithm allows the detection of more fluorophores than available channels *in vivo*

**Figure 5:**
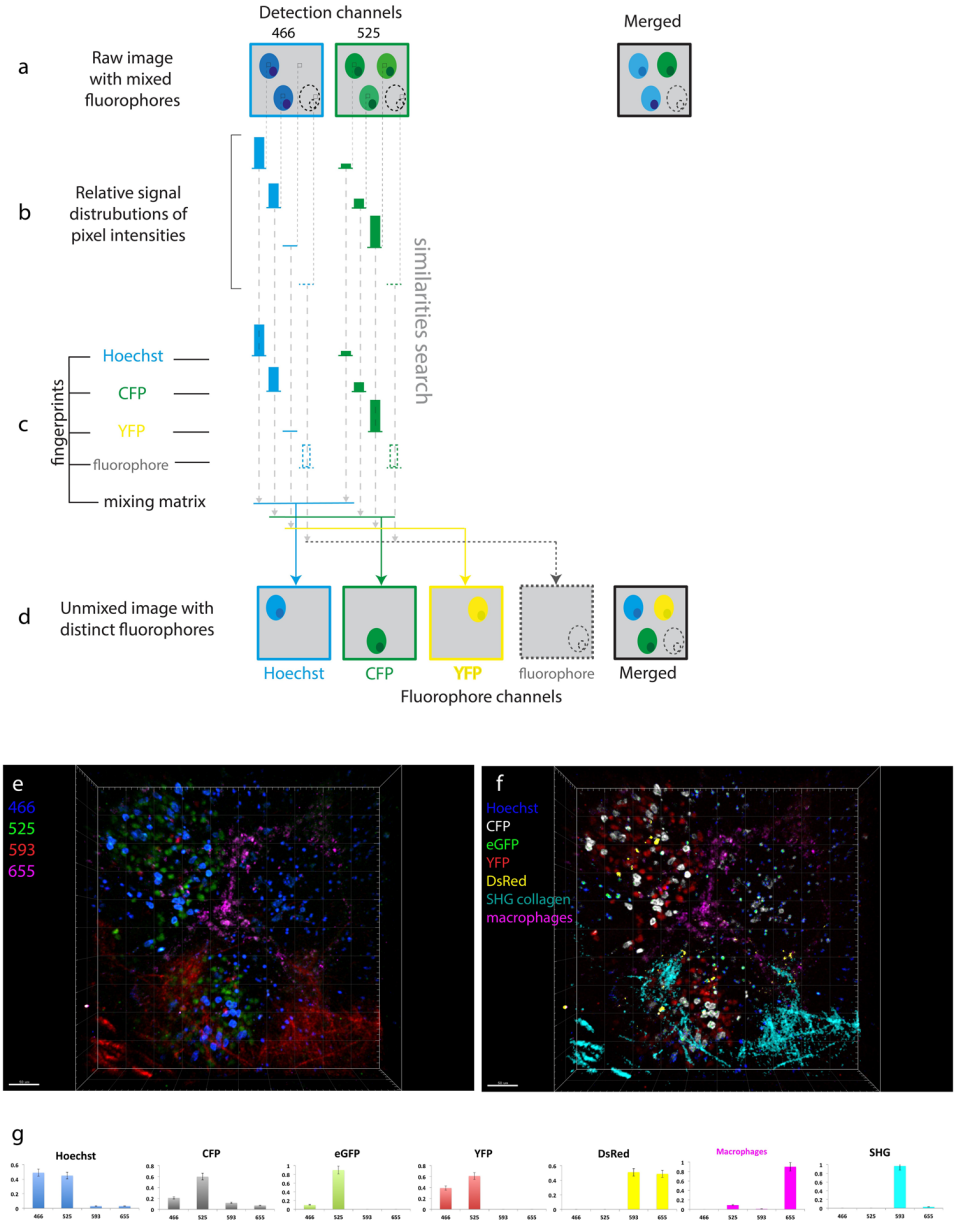
Similarity unmixing in the underdetermined case *in vivo*. (**a**–**d**) Schematic of the similarity algorithm in the underdetermined condition. (**a**) Raw images of cellular objects show crosstalk in two detection channels. The merged image illustrates color ambiguity of cells. The dashed contour represents a cell expressing any additional fluorophore. (**b**) The algorithm extracts a relative signal distribution from two channels pixels by pixel. The pixels containing the cellular fluorescent signal indicate signature of ambiguous fluorophores. (**c**) The fingerprints of Hoechst, CFP, YFP are measured at the single color condition. The algorithm searches for similarities between the signatures of undefined fluorophores from (**b**) and the known fingerprints channel by channel (dashed arrows). The key point for color separation in the underdetermined condition is the different ratios between the fingerprint elements of various fluorophores. The number of fluorophores is not limited unless the difference between their fingerprints is negligible (dashed bars of the fluorophore fingerprint). (**e**) Raw three-dimensional view of murine lymph node imaged on four channels. The lymphocytes are labeled with one of five fluorophores: Hoechst, CFP, hrGFP, YFP, DsRed. (**f**) Unmixed image of (**e**) containing seven color parameters (five fluorescent colors, second harmonic generation (SHG) from collagen and autofluorescence from macrophages). (**g**) The fingerprints of Hoechst, CFP, hrGFP, YFP, DsRed, macrophages and SHG. Scale bars, 50 μm.

In contrast to the conventional linear unmixing approach, the SIMI algorithm provides solutions in the underdetermined case, i.e. when the number of fluorophores exceeds the number of detection channels. The ability of our algorithm to separate chromophores in the underdetermined case is highlighted in Fig. 5 for the case of two detection channels and three chromophores, i.e. Hoechst, CFP and YFP, in which case the bleed-through between the detection channels leads to ambiguity of the fluorophores in the merged image (Fig. 5a). In the same manner as for the determined or overdetermined case, the SIMI algorithm extracts the normalized signal distribution ${b}_{1},{b}_{2}$ (Eq. 4) from each pixel (Fig. 5b), which is matched to the fluorophore fingerprints (Fig. 5b,c) by least square fitting (Eq. 6). Although the fingerprints contain only two elements ${b}_{1}\mathrm{and}\,{b}_{2}$, different ratios between these elements allow all three fluorophores to be distinguished (Fig. 5d). Theoretically, the number of fluorophores that can be separated from two or more detection channels is not limited (dashed cell in Fig. 5a,d). The larger the differences between the fluorophore fingerprints and the more elements these contain, i.e. the more detection channels are available, the better the quality of fluorophore unmixing. Concluding, the key feature of our algorithm is to separate fluorophores with different fingerprints, even if the number of detection channels is fewer than the number of fluorophores.

In order to demonstrate chromophore separation in the underdetermined case, under *in vivo* conditions, we applied the SIMI algorithm on images acquired in popliteal lymph nodes of anesthetized Rosa26^Confetti/Confetti^.Cre^ERT2^ mice^36^, using three detection channels (466 ± 20 nm, 525 ± 25 nm and 593 ± 20 nm). In these mice, three tissue compartments are labeled by six distinct markers: the nucleus of naïve B cells is labeled by Hoechst, lymphocytes express one of the four fluorescent proteins encoded in the Confetti allele (CFP, hrGFP, YFP or DsRed (Rosa26^Confetti/Confetti^.Cre^ERT2^ mouse strain) and collagen fibers display second-harmonic generation (SHG) (Fig. 5e,f). The fluorescent proteins are expressed at different locations in lymphocytes: CFP on the membrane, hrGFP in the nucleus and YFP and DsRed in the cytoplasm, and indicate the clonal relations of the cells after tamoxifen induction (3x, every 24 h). The strong spectral overlap of Hoechst, CFP and hrGFP shows both naïve B cells and other lymphocytes mainly in the blue channel. Only the distinct cellular location of the labelling gives a vague hint regarding the different fluorophores and their cellular origin (Fig. 5e). Similarly, both DsRed in lymphocytes and SHG from collagen fibers are mainly observed in the red channel (593 ± 20 nm) and strongly overlap. Using the difference in the individually acquired fingerprints (Fig. 5g), SIMI allows us to separate all six signals and, thus, to unequivocally identify all labeled tissue compartments (Fig. 5f). Additionally, using SIMI, we identified a unique fingerprint for macrophages, including tingible body macrophages, – based on their autofluorescence due to their phagocytic activity, and separate them as a seventh signal within the lymph node germinal centers (Fig. 5f).

### Imaging the dynamic orchestration of seven tissue compartments within germinal centers

**Figure 6:**
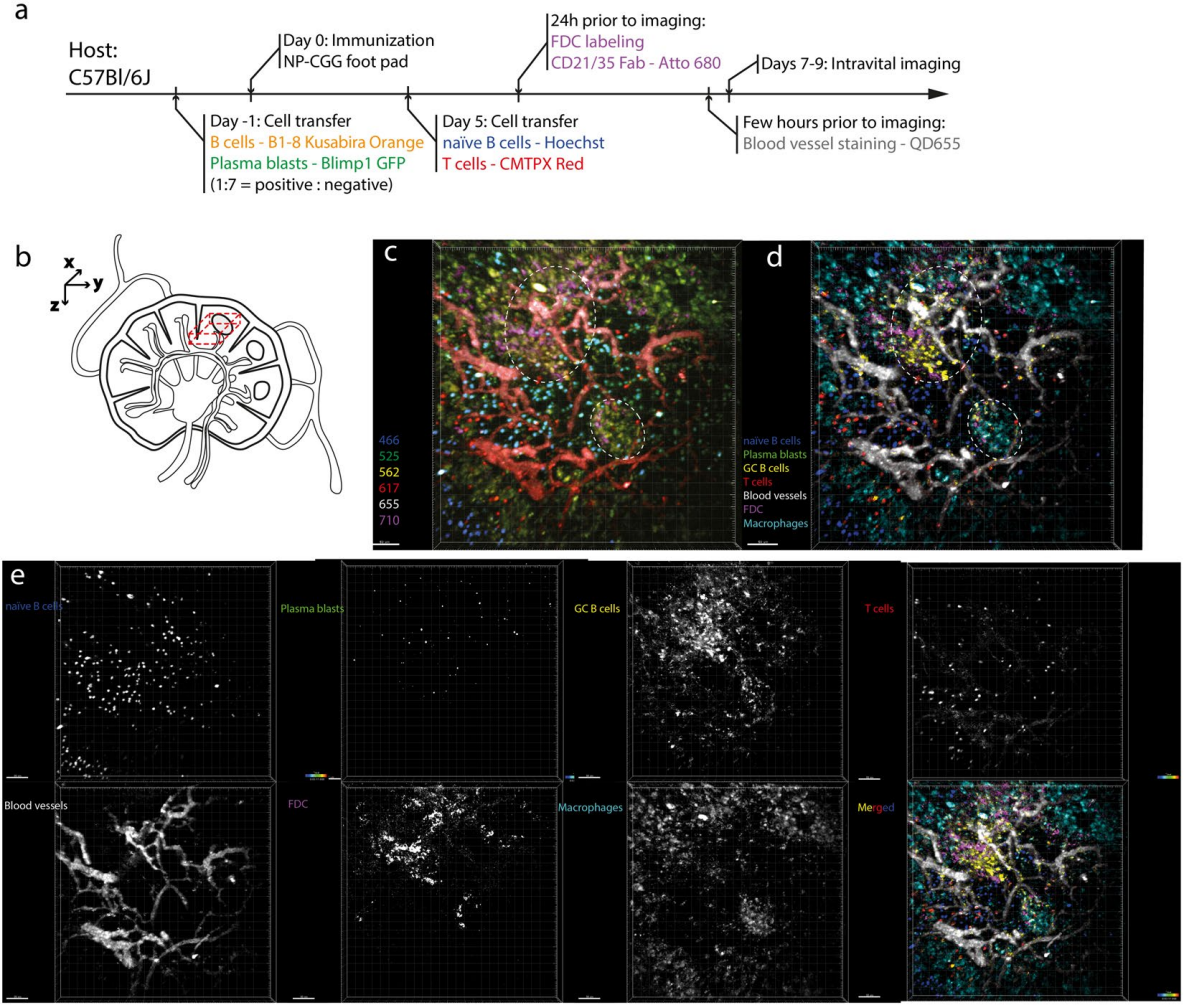
Dynamic multiplex *in vivo* imaging of germinal centers in the lymph node. (**a**) Preparation scheme of six-color mouse model. (**b**) Imaging region of popliteal lymph node (dashed 3D volume). (**c**) Raw 3D fluorescence images (500 × 500 × 40 µm³) in the popliteal lymph node of a recipient mouse prepared as described in (**a**) – NP-CGG immunization, imaging at day 7 after immunization. The time-lapse 3D images are recorded in six channels: 466 ± 20 nm (blue), 525 ± 25 nm (green), 562 ± 20 nm (orange), 617 ± 35 (red), 655 ± 20 (grey), 710 ± 20 (magenta). Dashed circles represent ongoing germinal centers identified by the accumulation of B1-8 cells and FDC cells. (**d**) SIMI-unmixed 3D fluorescence image of (**c**). The unmixed image contains seven distinct cellular and tissue compartments (Hoechst – naïve B cells, Blimp1+ plasma blasts – eGFP, GC B1-8 cells - Kusabira Orange, CD4+ T helper cells – CMTPX Red, blood vessels – QD655, FDCs CD21/35 – Atto 680, tingible body macrophages – autofluorescence due to phagocytosis). (**e**) Individual fluorophore channels of the seven tissue compartments in grey scale. Scale bars, 50 μm.

Successful validation of triple two-photon excitation combined with the SIMI approach on isolated splenocytes labeled by six different chromophores (Fig. 2c,d) allowed us to translate our method to multicolor intravital imaging of germinal center reactions in murine lymph nodes. In order to dynamically investigate the various cellular and tissue players during a well-defined germinal center reaction, we transferred NP-specific B cells isolated from B1-8^+/+^ Jκ^−/−^ Kusabira Orange × Blimp1-GFP mice into C57Bl/6 recipients. We immunized these mice with NP-CGG (Fig. 6a) according to previously published protocols^5, 37^ and performed intravital imaging of the popliteal lymph node between day 7 and 9 after immunization, when we expected the peak of germinal center reaction^10^. Five days post-footpad immunization, we transferred Hoechst labeled naïve B cells and CMTPX Red labeled CD4+ T cells. One day prior to imaging, we intravitally labeled follicular dendritic cells (FDC) with CD21/35-Fab-Atto680 injected s.c. into the footpad. Quantum dots (Qdots) 655 were injected intravenously in order to visualize the vasculature immediately prior to imaging. Using triple excitation in our two-photon microscope, we acquired six-channel time-lapse 3D images of germinal centers within B cell zones of popliteal lymph nodes (Fig. 6c). The challenge to distinguish between the individual chromophores (and between the individual cellular compartments) in this image is related not only to the spectral overlap of the emission spectra of the chromophores, but also by the additional autofluorescence of tingible body macrophages. These cells engulf whole or parts of cells labeled by different fluorophores, leading to a combination of various fluorophore signals, i.e. yellow and green areas in Fig. 6c. The autofluorescence in macrophages obstructs especially the identification of germinal center B cells (Kusabira Orange) and of plasma blasts (GFP). Only FDC networks, labeled with a near-infrared dye, indicate the position of two germinal centers (dashed circles, Fig. 6c).

We processed the raw image in Fig. 6c using the SIMI algorithm in the underdetermined case, i.e. six detection channels and seven distinct fluorescence signals. Next to the fluorophores used for labeling of specific cells or structures (Hoechst, eGFP, Kusabira Orange, CMTPX Red, QD655 and Atto680), the autofluorescence of the tingible body macrophages is considered as an additional signal. The autofluorescence of macrophages and fluorescence resulting from Qdots655 inside the vasculature were not recorded in isolated cells, thus, no fingerprints was acquired for these signals. Nevertheless, due to their individual structures and specific origin we could easily define their fingerprints directly from the raw intravital image (Suppl. Fig. 4). Additionally, the spectrally sharp signal of second harmonic generation (SHG) of collagen fibers constituting the conduits and the capsule of lymph nodes is the eighth signal distinguishable in our setup (not shown in Fig. 6). The spectrally unmixed 3D image clearly shows the two germinal centers, with labeled FDCs spatially correlating with the accumulation of Kusabira Orange-labeled GC B cells. Further, the unmixed image reveals the spatial distribution of naïve B cells, CD4+ T cells as well as of plasma blasts, the presence of macrophages within germinal centers and also in the cortical areas of the lymph nodes (Fig. 6d,e).

**Figure 7:**
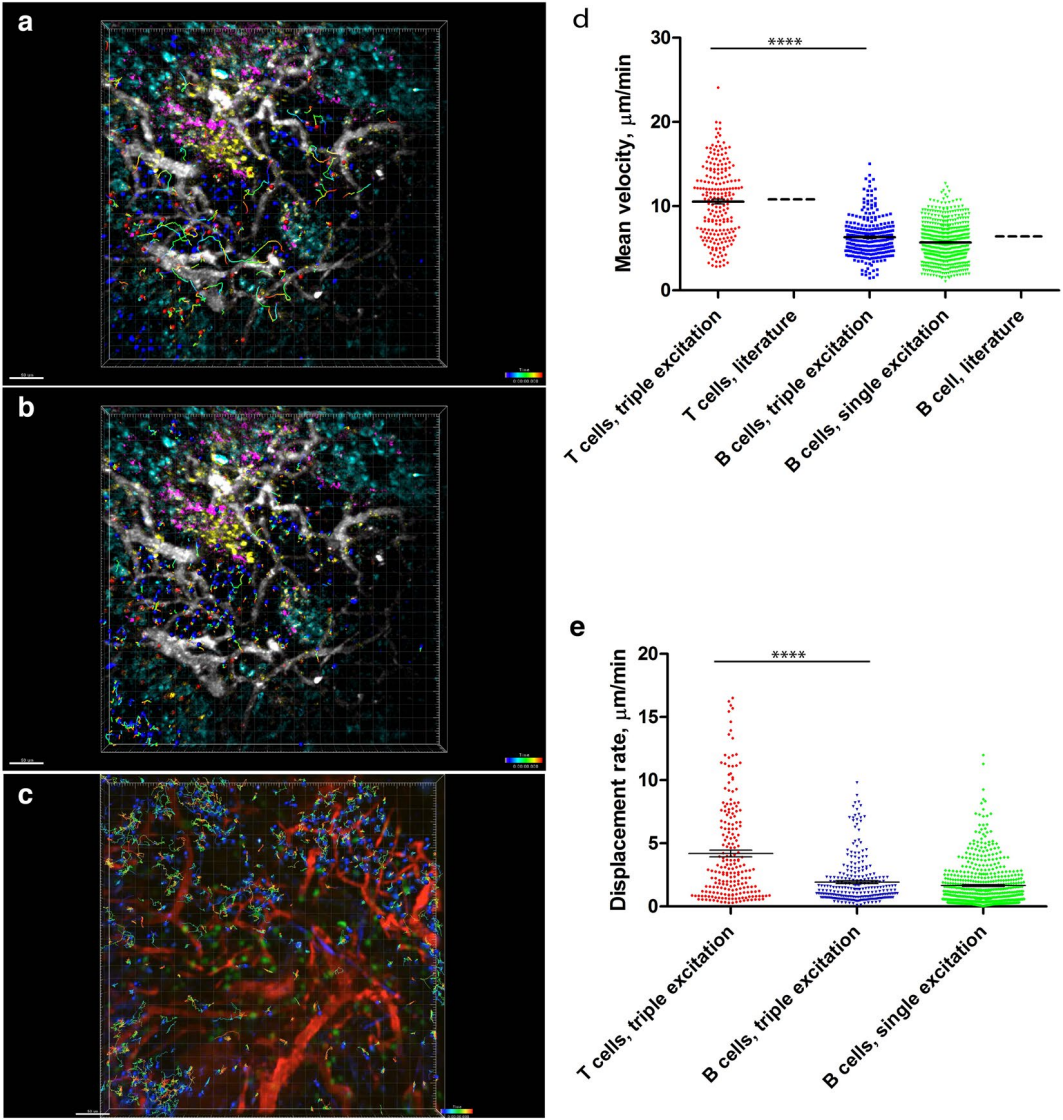
Naïve B and T helper cell motility patterns are similar in conventional and multiplex *in vivo* imaging, respectively, during GC reactions. (**a**,**b**) SIMI-unmixed time-lapsed 3D fluorescence images (500 × 500 × 40 µm³) as described in Fig. 6, with tracked CD4+ T and naïve B cells. The cell tracks are depicted as rainbow colored lines. We performed triple two-photon excitation at 850 nm (Ti:Sa), 1230 nm (OPO) and virtually at 1005 nm. (**c**) Time-lapsed 3D fluorescence images (500 × 500 × 30 µm³) in the lymph node of an anesthetized mouse, at day 7 after NP-CGG immunization. Naïve B cells labelled by Hoechst are depicted in blue, B1-8 GFP cells (GCs cells) are depicted in green and blood vessels labeled by rhodamine-dextran are shown in red. In this case, we performed a single two-photon excitation at 930 nm (Ti:Sa). Mean velocity distribution (**d**) and the displacement rate (**e**) of naïve B- and T helper cells, respectively, is significantly different, as previously reported. The absolute values of the mean velocity of both naïve B cells and T cells match the published values for B and T cell velocities^9^, depicted in graph (**d**). Additionally, both the mean velocity and the displacement rate of naïve B cells after triple two-photon excitation (with Ti:Sa and OPO), obtained from (**a**,**b**), are the same as the values measured after single two-photon excitation (with only Ti:Sa), obtained from (**c**), as indicated in the graphs (**d**) and (**e**). Mann-Whitney non-parametric tests were used for statistical analysis of the data. (*p < 0.05, **p < 0.01, ***p < 0.001, ****p < 0.0001). Scale bars, 50 μm.

Using the SIMI algorithm, we could also spectrally unmix time-lapse 3D fluorescence images of germinal centers (Suppl. Video 3). We could analyze the dynamics of all seven cellular and tissue compartments and could track in the same video both naïve B cells and the CD4+ T cells (Fig. 7a,b, Suppl. Videos 4 and 5) labeled with Hoechst and CMTPX red, respectively. In line with previous findings^9^, the CD4+ T helper cells show faster dynamics than the naïve B cells. We found significantly higher mean velocities and displacement rates of T cells (n = 165 cells) as compared with naïve B cells (n = 282 cells) (Fig. 6h,i). The mean velocity of T cells amounts to 10.55 ± 0.29 µm/min and of B cells to 6.30 ± 0.14 µm/min (s.e.m.), in agreement with previously measured values for naïve B cells and T helper cells^9^ as illustrated in Fig. 7d. The displacement rate of T cells amounts to 4.20 ± 0.27 µm/min, whereas that of B cells is 1.93 ± 0.11 µm/min (s.e.m.).

In order to quantify the tissue burden caused by two excitation lasers as compared to one excitation laser, we compared the motility of naïve B cells in the popliteal lymph node using our triple excitation approach (Fig. 7a,b) and a single-wavelength excitation approach (Ti:Sa tuned at 930 nm, photon flux 2.2·10^28^ photon/cm^2^·s, Fig. 7c, Suppl. Video 6). We found the same mean velocity (Fig. 7d) and the same displacement rate (Fig. 7e) of naïve B cells in both cases. The imaging time window was in both cases longer than an hour, with one 40 µm stack of images acquired every 15 s. Hence, we conclude that two excitation lasers (Ti:Sa and OPO) do not cause more cellular burden or damage than one laser (Ti:Sa). Moreover, we have previously used a neuronal Ca^2+^ reporter as an indicator of cellular dysfunction for prolonged imaging. Upon neuronal damage, their intracellular Ca^2+^ levels increase, which is visible through Förster resonance energy transfer (FRET). We imaged 3D stacks of 300 × 300 × 50 µm³ every minute, over two hours at similar laser powers as described here, using dual excitation (Ti:Sa at 850 nm and OPO at 1100 nm), with no increase in neuronal calcium, i.e. no signs of neuronal dysfunction^12^.

## Discussion

In the last decade, *in vivo* imaging in secondary lymphoid organs has revealed unique insights into the dynamics and cellular communication during immune responses^5, 9, 10, 38^. With regard to the germinal center reaction, imaging has helped to place key stages in B cell differentiation as well as the initiation of immunological memory in the genuine, *in vivo* context. Still, while germinal center reactions as well as immune responses, in general, are influenced by the interplay of multiple cellular subsets and tissue compartments, conventional intravital microscopy is limited to a maximum of four colors, i.e. four distinguishable cellular and tissue compartments. In order to understand the dynamics and interactions of all contributing players *in vivo*, a multicolor intravital imaging approach is needed. While pointing in this direction, the approach of Mahou *et al*.^13^ demonstrates by wave-mixing of OPO and Ti:Sa lasers only four-color dynamic deep-tissue imaging (two fluorescence signals, in addition to second and third harmonics), which is not sufficient to investigate the complexity of immune or neuronal responses *in vivo*.

We present in this work a synergistic strategy for multicolor *in vivo* imaging with three major components: wavelength mixing for simultaneous triple two-photon excitation of all components, a wide range of fluorophores from blue to near infrared, and an effective spectral unmixing approach using SIMI, our novel similarity unmixing algorithm. The combination of these methods allowed us to overcome the color limitation of current intravital imaging techniques and to monitor the interplay of dynamic cellular processes at a new level of complexity during germinal center reactions *in vivo*.

The wavelength mixing two-photon excitation is a promising approach with appealing features. Unlike sequential single-laser excitation^14^, it provides the simultaneous and effective excitation of many fluorophores that is required for imaging of dynamic processes. Our synchronized two-laser approach simultaneously enables three distinct excitation opportunities, two symmetric two-photon excitations and an asymmetric two-photon excitation. It simplifies the alignment procedure as compared to the use of a three-laser system^11^ by requiring the alignment of only two lasers.

The use of either supercontinuum (SC) generation and fast wavelength-switchable systems as a multiphoton excitation source may be a good alternative to multiple femtosecond-pulsed conventional laser systems for certain applications, but for several reasons these optical sources have not found yet broad application in biosciences and biomedicine. Having an extremely broad bandwidth, SC sources suffer from serious chromatic aberration, which leads to poor optical sectioning and low contrast^22^. Moreover, the lack of independent power control of different spectral ranges of both the SC generation and the fast switchable laser systems makes them unsuitable for the simultaneous excitation of fluorophores with strongly different excitation and emission properties, for which an individual power adjustment is required. The SC platforms based on coupling of femtosecond pulses into nonlinear PCF show often low spectral power density that limits their application in multi-photon microscopy, especially deep in tissue^18^. Successful applications of both ns, ps and fs SC systems as well as rapid wavelength-switchable systems for in *in vivo* multi-photon microscopy, as compared to conventional femtosecond-lasers, still need to be demonstrated.

Moreover, the palette of fluorophores and fluorescent proteins used in cell biology now spans the spectral range from deep blue to near infrared^33, 39^. We selected fluorophores covering the whole range of this available spectrum, and chose them in order to minimize spectral overlap. This strategy allowed us to increase the number of colors, which label multiple cellular and tissue players in secondary lymphoid organs, during germinal center reactions *in vivo*.

Still, the major bottleneck of simultaneous multiplexed fluorescence imaging is the ability to distinguish between multiple fluorophores. The crosstalk originating from spectral overlap of neighboring fluorophores hinders the unambiguous identification of different cell types in complex dynamic processes. In order to separate distinct colors labeling up to seven different cell and tissue compartments in the popliteal lymph node, we developed and applied the similarity-unmixing algorithm SIMI. SIMI shows distinct advantages over conventional spectral unmixing techniques. Unlike spectral deconvolution, which identifies only the reference channel (the channel with the highest intensity)^14^, it takes information from all detection channels, for each fluorophore into account. For this reason, we are able to distinguish those fluorophores, which have the same reference channel but different signatures in other channels. Further, the SIMI algorithm is independent from crosstalk relations between the mixing signals, in contrast to the subtraction approach^11^, in which the color discrimination is possible only for the independent crosstalk of mixed pairs. In spectrally multiplexed images, the number of detection channels often limits the number of fluorophores that can be simultaneously resolved. This restriction represents the underdetermined case, for which the widely used linear unmixing fails but our similarity algorithm remains fully functional. An appealing alternative to SIMI is the use of a phasor unmixing approach, recently demonstrated in one-photon excitation confocal microscopy of zebra fishes^40^. Since Fourier transforms needed in this approach are very sensitive to signal noise, its use has to be verified on the rather noisy emission signals originating from optically non-linear processes, like in our case.

In conclusion, simultaneous triple two-photon excitation based on wavelength mixing in combination with an extended set of fluorophores towards the near infrared opens new horizons for intravital imaging of dynamic processes in the immune system. Further, our technology opens new perspectives of functional analysis in the genuine context, if combined with FRET approaches to probe molecular parameters, and with Confetti or Brainbow mouse lines, e.g. to analyze clonal relations and competition of antigen-specific B cells during immune responses^38^. We demonstrated here that the SIMI approach is able to solve underdetermined detection problems in the case of single chromophore labeling. However, the use of our approach is not limited to such applications: to use SIMI for the analysis of FRET data, it must be extended to compare the detection signal per pixel with the linear combination of two fingerprints, i.e. of the donor and acceptor^41^. The FRET ratio results from the coefficients of the linear combination. We expect that in the future our strategy will shed light on the complexity of spatiotemporal dynamic interplay of various cell types involved in germinal center reactions, in immune niches within the bone marrow, and chronic or acute tissue inflammation of other organs, in addition to applications in neurosciences and developmental biology.

## Material and Methods

### Two-photon laser-scanning microscope setup

Two-photon fluorescence imaging experiments were performed as previously described^42^, using a specialized laser-scanning microscope based on a commercial scan head (TriMScope II, LaVision BioTec, Bielefeld, Germany). A near-infrared laser (Ti:Sa, Chameleon Ultra II, Coherent, Dieburg, Germany) and an infrared laser (OPO, APE, Berlin, Germany) were used as excitation sources. The Ti:Sa and OPO beams, both linearly polarized, were combined in the scan head using a dichroic mirror (T1045, Chroma, US). A water-immersion objective lens (20x, NA 1.0, Plan-Apochromat, Carl Zeiss, Jena, Germany) was used to focus both laser beams into the sample. The laser pulse trains were temporally synchronized using a piezo-motorized delay stage (MS30, Qioptiq, Göttingen, Germany), while the relative divergence of the two lasers was controlled by beam expanders. The laser power was controlled by combinations of λ/2 wave-plates and polarizers. The ultrashort pulses of both lasers were compressed using external compressors: a commercial two-prism-based compression for the Ti:Sa beam and a home-built single-prism compressor for OPO. Fluorescence, SHG, SFG and wavelength mixing signals were collected in the backward direction using dichroic mirror (775, Chroma, US) and directed to six photo multiplier tubes (H7422, Hamamatsu, Japan). All PMTs were assembled in a detection system with different optical channels, where every channel was determined by individual fluorescence filter and a set of dichroic mirrors as indicated in the manuscript: 466 ± 20 nm, 525 ± 25 nm, 562 ± 20 nm, 593 ± 20 nm, 617 ± 35 nm, 655 ± 20 nm and 710 ± 20 nm. In all imaging experiments we used an average maximum laser power of 10 mW to avoid photodamage. The acquisition time for an image with a field-of-view of 500 µm × 500 µm and a digital resolution of 1024 × 1024 pixel was 944 ms. We acquired 40 µm z-stacks (z-step 2 µm) every 20 s over a total time course of typically 30 minutes.

### Data analysis

Image segmentation and tracking of all cells were performed using existing segmentation, object-recognition and tracking plugins in Imaris (Bitplane, UK). Statistical analysis of the data was performed using Graph Pad Prism. The SIMI algorithm was integrated as PlugIn in the linear unmixing PlugIn of Fiji/ImageJ written by Joachim Walter. The custom-written code is available from the authors upon request.

### HEK cells transfection and imaging

We prepared two types of isolated HEK cell samples. First, we prepared samples containing HEK-293T cells expressing a single color of one out of the five FPs. For each single–labeled fluorophore, we acquired images on all six PMT channels and extracted a fingerprint, also known as a signature, of a given fluorescent protein. The fingerprint represents a ratio of relative intensities in different PMT channels and serves as the main criterion in our spectral unmixing analysis. Second, we prepared samples containing a mixture of single-labeled HEK-293T cells, each expressing one of five FPs. To achieve ‘one cell – one color’ labeling in the sample mixture first we transfected HEK-293T cells separately with different FP-encoding vectors, and then mixed these cells in equal proportions on one collagen-coated plate. We transfected HEK cells following the protocol provided for Lipofectamine 3000 (ThermoFischer Scientific, Waltham, MA), using vectors encoding eCFP, eGFP, mOrange2, mKate2 (Addgene, Cambridge, MA) and eqFP670 (Evrogen, Moscow, Russia).

### Splenocyte isolation, labelling and imaging

We isolated splenocytes from the spleen of C57Bl/6 mice and prepared, similarly to the HEK cells, two types of isolated splenocyte samples: single color samples to acquire the spectral signatures of the chromophores and mixed samples. For the cell isolation, the spleen was cut into small pieces, pressed through a strainer and suspended in RPMI medium containing 10% FCS. Erythrocyte lysis buffer was added to the cell suspension to remove erythrocytes. The suspension was centrifuged and the pellet was resuspended in PBS. The labeling of the splenocytes was performed following existing protocols, using Hoechst, CMTPX Red, Alexa647 (ThermoFischer Scientific, Waltham, MA) and Atto680 (AttoTEC, Siegen, Germany). Splenocytes from B1-8^+/+^ Jκ^−/−^ Kusabira Orange mice as well as of mice ubiquitously expressing eGFP were isolated from the spleen in a similar manner as described for C57/Bl6 mice.

### Mice

All mice used were on a C57Bl/6 background. We used B1-8^+/+^ Jκ^−/−^ Kusabira Orange x Blimp1-GFP mice, generated by crossing of B1-8^+/+^ Jκ^−/−^ mice (kindly provided by K. Rajewski and A. Haberman) with Blimp1-GFP mice. Other experiments used F1 mice from a breeding of Rosa26-Brainbow2.1 mice^36^ (obtained from Jackson Laboratories) with Rosa26-Cre^ERT2^ mice^43^ (obtained from Taconic). All animal experiments were approved by Landesamt Für Gesundheit und Soziales, Berlin, Germany in accordance with institutional, state and federal guidelines.

### Mouse immunization strategy

In order to a defined model of germinal center reaction in the popliteal lymph node, we immunized mice according to the scheme in Fig. 3a, as previously described^5, 37^. Therefore, we isolated B cells from B1-8^+/+^ Jκ^−/−^ Kusabira Orange x Blimp1-GFP mice and mixed them with non-fluorescent B cells from non-fluorescent B1-8^+/+^ Jκ^−/−^ mice at a ratio of 1:7. We transferred intravenously 10^6^ isolated B cells per animal into recipient C57Bl/6 mice, one day prior to immunization with NP-CGG. In addition we isolated naïve B cells and CD4+ T cells from C57Bl/6 mice, stained them with Hoechst and cell tracker CMTPX Red, respectively, and transferred 3·10^7^ B cells and 3·10^7^ T cells per mouse into the recipients, five days after immunization. We injected CD21/35-Fab antibodies conjugated with Atto680 in the food pad of recipient mice 24 hours prior to imaging, in order to label the FDC network. The vasculature of the recipient mice was labeled with QD655 immediately before intravital imaging. We intravitally imaged the popliteal lymph node of the mice between day 7 and day 9 after immunization, corresponding to the peak of the germinal center reaction.

### Surgical preparation of the popliteal lymph node for intravital imaging

The preparation of popliteal lymph nodes for intravital imaging was performed as previously reported^5^. Mice were anaesthetized by i.p. injection of ketamin/xylazin, according to their weight. Reflexes were tested to monitor the depth of anesthesia over the entire imaging period. The anaesthetized mouse was transferred to a custom-built surgery and microscopy stage and fixed with dedicated tweezers. The popliteal lymph node was exposed, kept moist using isotonic 0.9% NaCl and covered with a glass cover slip of 0.13 mm thickness. A temperature of 37 °C was maintained at all times during imaging using a heating coil, and the body temperature was also maintained at 37 °C with a specialized heating foil placed under the animal. After each imaging experiment, mice were sacrificed.

### Recording of two-photon spectra of various chromophores

In order to ensure optimal triple excitation of the chosen chromophores, both fluorescent proteins and dyes, we measured their two-photon excitation spectra in cells, as we previously described^12^ (Fig. 2b,d; upper panels). Spectra were recorded in live cells for a better comparability with the *in vivo* situation and were measured in a wide wavelength range by the means of Ti:Sa (760 ≤ λ_Ti:Sa_ ≤ 1040 nm) and OPO (1060 ≤ λ_OPO_ ≤ 1300 nm). To achieve a continuous two-photon spectrum, the row data were corrected for background signal and peak photon flux, which includes squared laser power (measured simultaneously by reflecting about 4% of laser beams into a photodiode), photon energy in pulse peak, pulse width in focus (measured by external auto-correlator), repetition rate of lasers and excitation volume at each excitation wavelength. To avoid saturation and to support two-photon process we kept the laser power at moderate values.

### Data availability

The data that support the findings of this study are available from the corresponding author upon request.

## Electronic supplementary material

Supplementary information

Suppl Video1

Suppl Video2

Suppl Video3

Suppl Video4

Suppl Video5

Suppl Video6
