# Supplementary material for: Synergistic Strategy for Multicolor Two-photon Microscopy: Application to the Analysis of Germinal Center Reactions In Vivo
Source: Sci Rep. 2017 Aug 2;7:7101. doi: 10.1038/s41598-017-07165-0 (PMC5540998; doi:10.1038/s41598-017-07165-0)
Supplement: Supplementary file 1 — Supplementary information [file 41598_2017_7165_MOESM1_ESM.pdf]

SYNERGISTIC STRATEGY FOR MULTICOLOR TWO-PHOTON MICROSCOPY: APPLICATION TO THE ANALYSIS OF GERMINAL CENTER REACTIONS IN VIVO

ASYLKHAN RAKHYMZHAN, RUTH LEBEN, HANNA ZIMMERMANN, ROBERT GÜNTHER, PEGGY MEX, DAVID REISMANN, CAROLIN ULBRICHT, ANDREAS ACS, ALEXANDER U. BRANDT, RANDALL L. LINDQUIST, THOMAS H. WINKLER, ANJA E. HAUSER, RALUCA A. NIESNER

SUPPLEMENTARY FIGURES

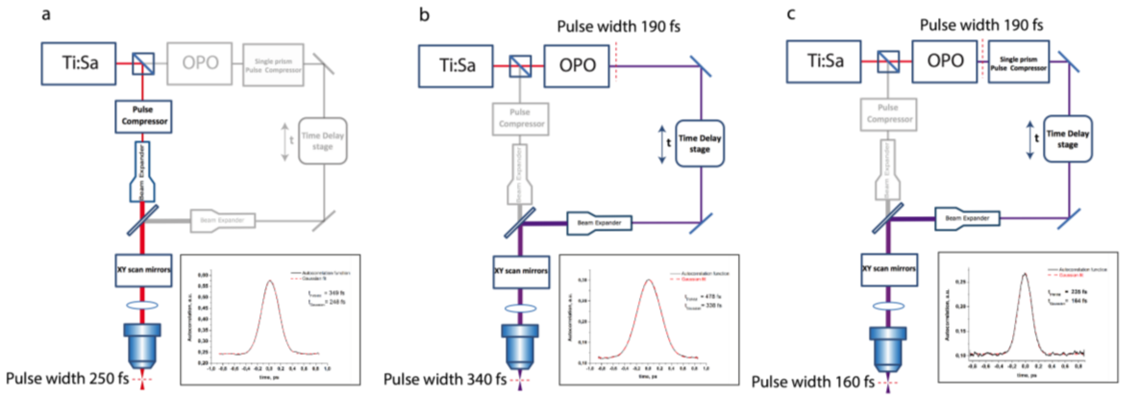

FIGURE S1

PULSE WIDTH OF TI:SA AND OPO LASERS IN THE MICROSCOPE WITH AND WITHOUT PULSE COMPRESSION. The pulse width of both Ti:Sa and OPO was measured using an optical auto-correlator (APE, Berlin, Germany), based on the second harmonic generation of birefringent KDP powder in the two-photon microscope. (a) The Ti:Sa pulse width was measured only with a conventional "two-prism" pulse compressor. (b, c) The OPO pulse width was measured without and with a single-prism pulse compressor, respectively. The pulse compression of the OPO beam was achieved using the single-prism design described in Fig. 1b. Having all positive features of two-prism configuration, the single-prism design offers many advantages over conventional prism-based pulse compressors. Significantly simplified alignment is achieved due to the

corner cube optical properties of anti-parallel beam reflection and precise compensation of the spatiotemporal distortions (angular and spatial dispersion, pulse-front-tilt) in the output beam. It is also very compact because the beam double-passes the prism corner cube path, which is half the size of the two-prism design. The wavelength tuning is achieved by rotation only one prism, unlike in the two-prism configuration, where proper angles of two prisms have to be maintained.

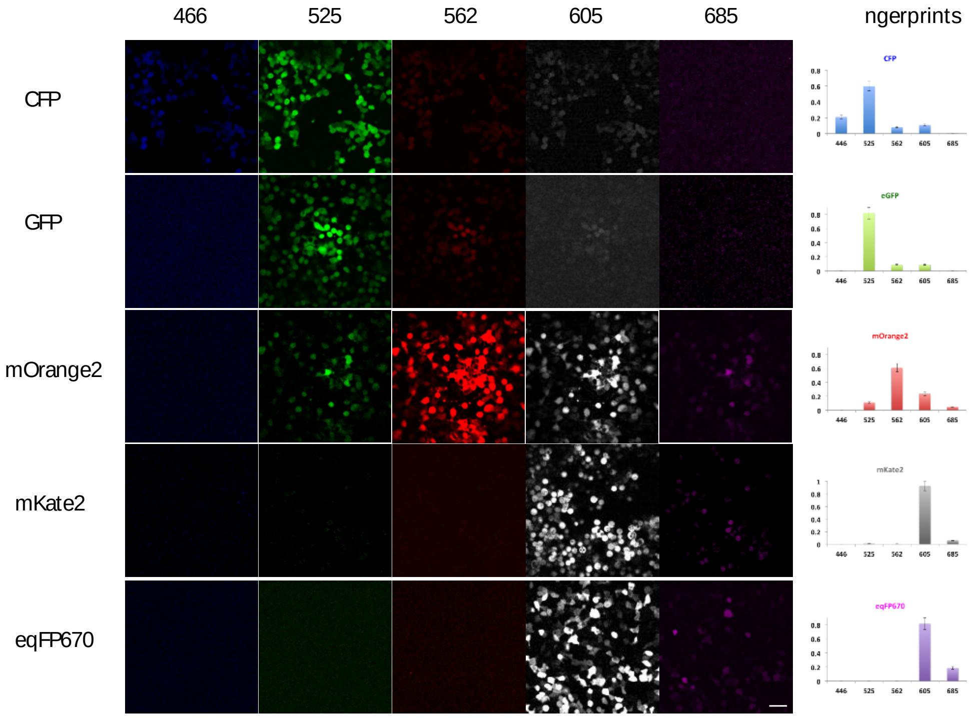

FIGURE S2

FINGERPRINT ASSESSMENT IN SINGLE-COLOR CONTROLS OF HEK CELLS. The single-color controls of isolated HEK cells were excited at 850 nm, 1230 nm, by spatially overlapped wavelengths with pulse trains synchronization. Their fluorescence signal was detected on five PMTs, as previously described (Fig. 1a). Each row illustrates images of HEK cells on five detection channels corresponding to one of the fluorescent proteins with its fingerprint. Scale bar, 50 μm.

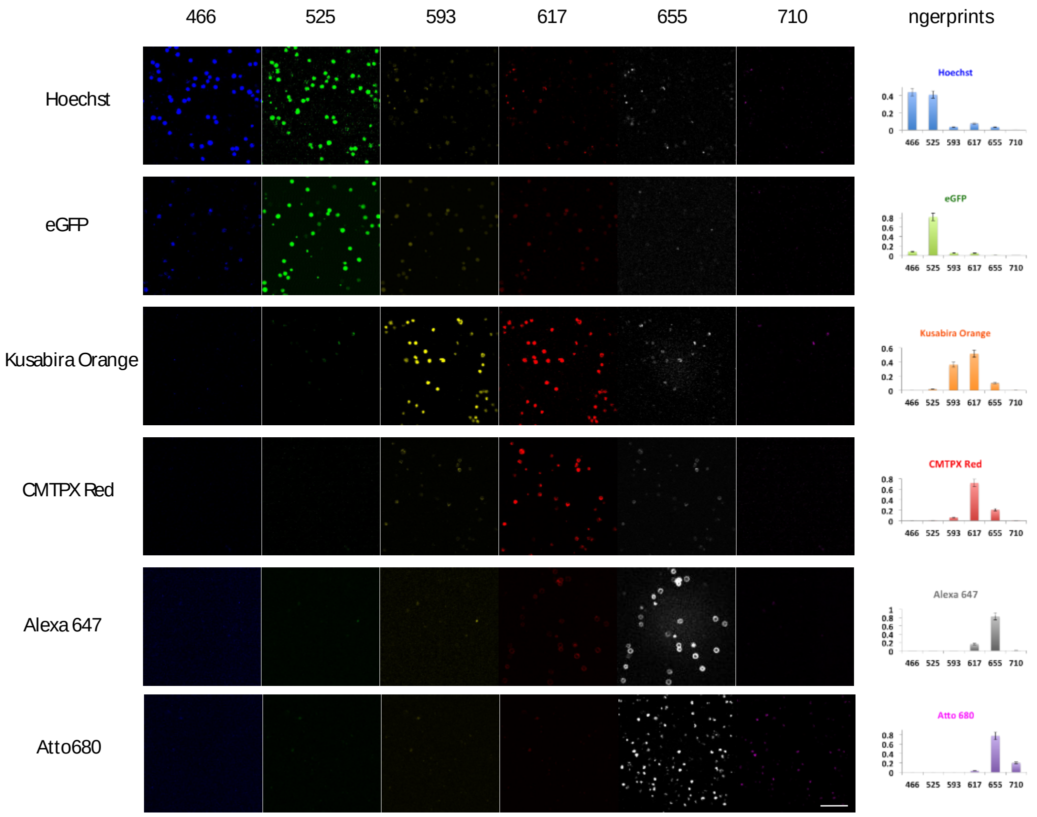

FIGURE S3

FINGERPRINT ASSESSMENT IN SINGLE-COLOR CONTROLS OF SPLENOCYTES. The single-color controls of isolated splenocytes were excited at 850 nm, 1230 nm, by both spatially overlapped wavelengths with pulse trains synchronization. Their fluorescence signal was detected on six PMTs, as previously described (Fig. 1a). Each row illustrates images of splenocytes on six detection channels corresponding to one of the fluorophores with its fingerprint. Scale bar, 50 μm.

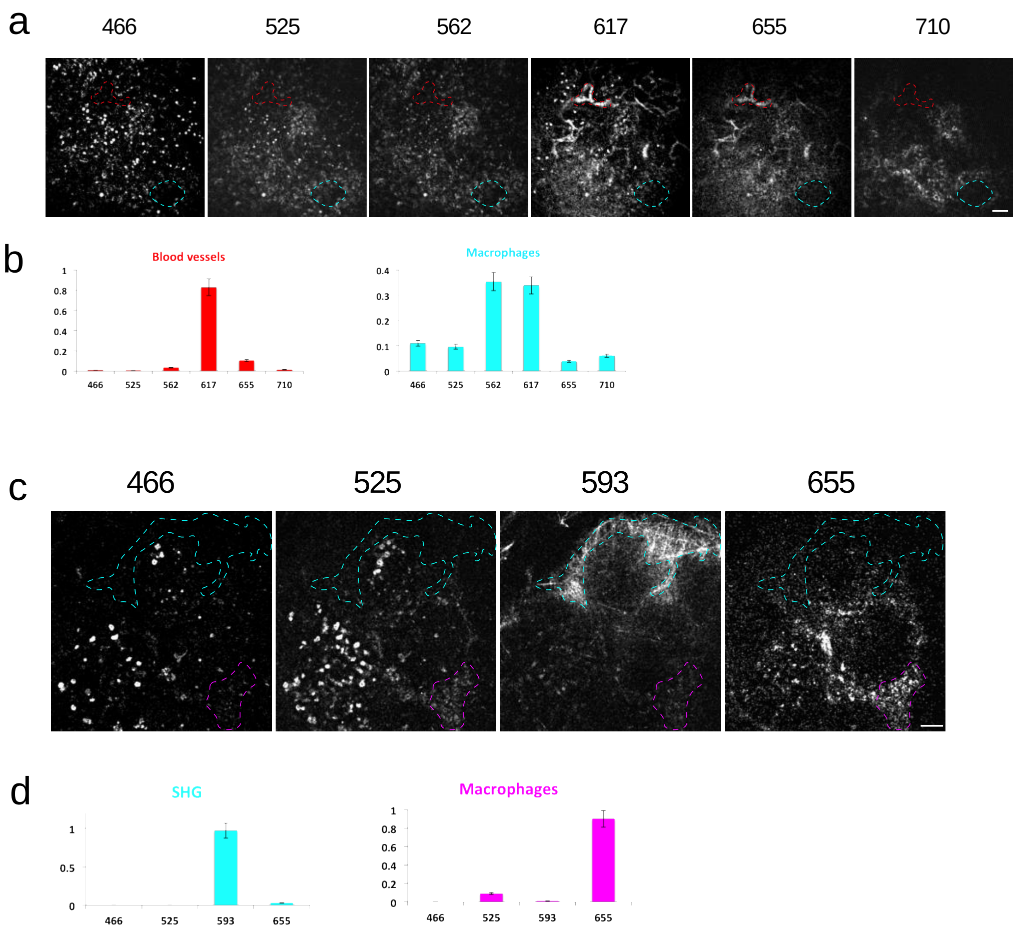

FIGURE S4

IN SITU ACQUIRED FINGERPRINTS OF THE SIGNALS USED FOR INTRAVITAL IMAGING OF GERMINAL CENTER REACTIONS AND FOR THE UNDERDETERMINED CASE *IN VIVO*. (a, b) Raw data and fingerprints of machrophages and blood vessels from intravital measurement of popliteal lymph node (Fig. 6c). Macrophages, including tingible body macrophages, show a autofluorescent signal in the intravital raw data, which helped us to generate their specific fingerprint from the dashed area (colored cyan), containing only the macrophages autofluorescent signal, and ultimately, to resolve them from other cellular compartments in lymph node imaging. The fingerprint of blood vessels, stained by quantum dots 655, was also acquired *in situ*, since its structure can be easily determined from the raw data (dashed area colored red). (c, d) Raw data and fingerprints of SHG from collagen fibers and machrophages from in vivo measurement of popliteal lymph node at the underdetermined condition (Fig.

5e). Signals from SHG and macrophages can be easily identified on the raw data (dashed areas colored cyan and magenta, respectively). The SHG signal appears in only one detection channel. Scale bars, 50 μm.

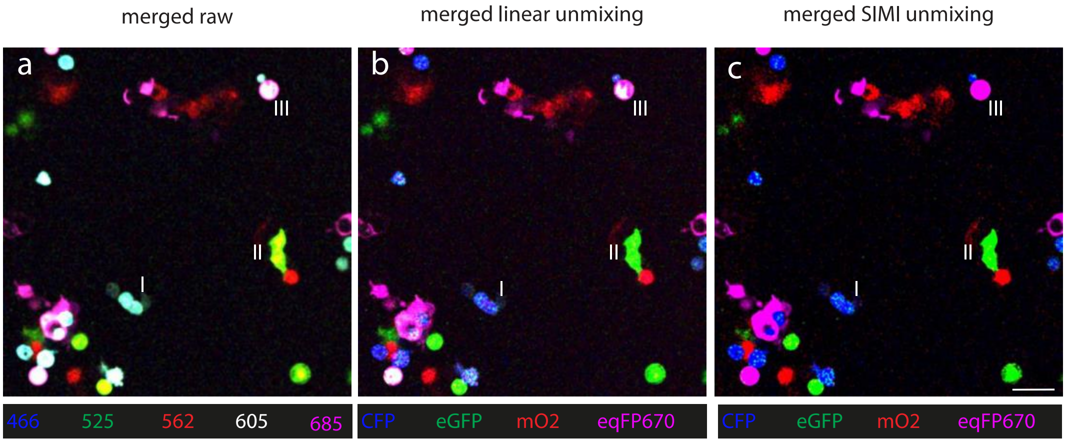

FIGURE S5

COMPARISON OF LINEAR UNMIXING AND SIMI ALGORITHM ON HEK CELLS IMAGE.

(a) Merged raw image of HEK cells mixture expressing four fluorescent proteins: CFP, eGFP, mOrange2 and eqFP670. Some cells, examples denoted by I, II and III, show a significant crosstalk on five channels. (b) Merged image processed by linear unmixing approach. The cases I and II, assigned as CFP and eGFP, respectively, demonstrate full color unmixing. The linear unmixing was incapable to determine the case III cells. (c) Merged image processed by SIMI algorithm. All cases, I, II and III, were completely assigned as CFP, eGFP and eqFP670, respectively. The SIMI algorithm demonstrates higher spectral unmixing capacity in compare with the linear unmixing approach. Scale bar, 50 µm.

MOVIE CAPTIONS

S VIDEO1

DEPENDENCE OF SUM FREQUENCY GENERATION IN POTASSIUM DIHYDROGEN PHOSPHATE (KDP) POWDER ON THE OVERLAP OF TI:SA AND OPO PULSE TRAINS.

(a) SHG signal from 850 nm on blue channel (447±30 nm). (b) Sum frequency generation of OPO (1230 nm) and Ti:Sa (850 nm) lasers in birefringent KDP powder is detected on green channel (525±25) at 502 nm only if their pulse trains overlap and disappears if the pulse trains are shifted with respect to each other, thus, generating a cross-correlation signal of the two lasers. (c) The OPO pulse train moves with respect to the Ti:Sa pulse train by changing the optical path length of the OPO beam into the microscope. Therefore, a delay stage consisting of two 90° prisms is used, one of the prisms being mounted on a piezo stage allowing for its translation as shown in Fig. 1a. The step of the piezo motor is 15 nm, corresponding to 50 attoseconds. (d) SHG signal from 1230 nm on red channel (617±35 nm).

S VIDEO2

DEPENDENCE OF MORANGE2 FLUORESCENCE SIGNAL EXPRESSED BY HEK CELLS ON THE OVERLAP OF TI:SA AND OPO PULSE TRAINS. Similar to SFG, the mOrange2 fluorescence can be detected at 562 nm only if the pulse trains of Ti:Sa and OPO overlap and disappears if the pulse trains are shifted with respect to each other (inset video), thus generating a cross-correlation signal of the two lasers.

S VIDEO3

INTRAVITAL SPECTRALLY MULTIPLEXED IMAGING OF A GERMINAL CENTER. Left: raw video. Right: video processed by SIMI algorithm. Time-lapse 3D fluorescence imaging of germinal centers (500x500x40 μm^3) in the popliteal lymph node of a mouse immunized with NP-CGG recorded after triple wavelength mixing excitation at 850 nm (Ti:Sa) and 1230 nm (OPO). The signals of naïve B cells (Hoechst, blue), germinal center B1-8 cells (Kusabira Orange, yellow), plasma blasts (Blimp1 GFP, green), CD4+ follicular T helper cells (CMTPX, red), follicular dendritic cells (CD21/35-Fab-ATTO680, magenta), blood vessels (QD655, grey) and macrophages including tingible body macrophages (autofluorescence, cyan) could be detected and resolved over time. 3D images were recorded every 20 s, the z-step was 2 µm.

S VIDEO4

INTRAVITAL SPECTRALLY MULTIPLEXED IMAGING OF A GERMINAL CENTER – TRACKING CD4+ T CELLS. Time-lapse 3D fluorescence imaging of germinal centers (500x500x40 μm^3) in the popliteal lymph node of the same mouse immunized with NP-CGG recorded after triple wavelength mixing excitation at 850 nm (Ti:Sa) and 1230 nm (OPO). The colored track paths shown in the right video indicate the tracks of CD4+ T helper cells.

S VIDEO5

INTRAVITAL SPECTRALLY MULTIPLEXED IMAGING OF A GERMINAL CENTER – TRACKING NAIVE B CELLS. Time-lapse 3D fluorescence imaging of germinal centers (500x500x40 μm^3) in the popliteal lymph node of the same mouse immunized with NP-CGG recorded after triple wavelength mixing excitation at 850 nm (Ti:Sa) and 1230 nm (OPO). The colored track paths shown in the right video indicate the tracks of naive B cells.

S VIDEO6

INTRAVITAL SINGLE EXCITATION IMAGING OF A GERMINAL CENTER – TRACKING NAIVE B CELLS. Time-lapse 3D fluorescence imaging of germinal centers (500x500x40 μm^3) in the popliteal lymph node of the B1-8 Jk-/- GFP mouse immunized with NP-CGG recorded after single excitation at 930 nm (Ti:Sa). The colored track paths shown in the left video indicate the tracks of naive B cells. The signals of naïve B cells (Hoechst, blue), germinal center B cells (GFP, green), blood vessels (rhodamin dextran, red) could be detected and resolved over time. 3D images were recorded every 20 s, the z-step was 2 μm.
